# Reversible splenial lesion syndrome associated with lobar pneumonia: Case report and review of literature

**DOI:** 10.1097/MD.0000000000004798

**Published:** 2016-09-30

**Authors:** Chunrong Li, Xiujuan Wu, Hehe Qi, Yanwei Cheng, Bing Zhang, Hongwei Zhou, Xiaohong Lv, Kangding Liu, Hong-Liang Zhang

**Affiliations:** a Neuroscience Center, Department of Neurology; b Department of Radiology, the First Hospital of Jilin University, Jilin University, Changchun, China.

**Keywords:** corpus callosum, magnetic resonance imaging, multiple sclerosis, reversible splenial lesion syndrome

## Abstract

**Background:**

Reversible splenial lesion syndrome (RESLES) is a rare clinico-radiological disorder with unclear pathophysiology. Clinically, RESLES is defined as reversible isolated splenial lesions in the corpus callosum, which can be readily identified by magnetic resonance imaging (MRI) and usually resolve completely over a period of time. RESLES could be typically triggered by infection, antiepileptic drugs (AEDs), poisoning, etc. More factors are increasingly recognized.

**Methods and results:**

We reported herein an 18-year-old female patient with lobar pneumonia who developed mental abnormalities during hospitalization. An isolated splenial lesion in the corpus callosum was found by head MRI and the lesion disappeared 15 days later. Based on her clinical manifestations and radiological findings, she was diagnosed with lobar pneumonia associated RESLES. We further summarize the up-to-date knowledge about the etiology, possible pathogenesis, clinical manifestations, radiological features, treatment, and prognosis of RESLES.

**Conclusion:**

This report contributes to the clinical understanding of RESLES which may present with mental abnormalities after infection. The characteristic imaging of reversible isolated splenial lesions in the corpus callosum was confirmed in this report. The clinical manifestations and lesions on MRI could disappear naturally after 1 month without special treatment.

## 1 Introduction

In 1999, Kim et al^[1]^ reported 6 patients with an undescribed-ever, reversible abnormality in the splenium of the corpus callosum (SCC) in epileptic patients receiving antiepileptic drugs (AEDs). These discrete, focal, ovoid, and nonhemorrhagic lesions were reversible and were considered to be related to the toxicity of AEDs.^[1]^ Later on, this imaging phenomenon was found not only in patients with epilepsy or patients receiving AEDs, but also in a few patients with encephalitis/encephalopathy caused by different viruses.^[23]^ In 2004, Tada et al^[2]^ reported a series of 15 patients with identical clinical characteristics and clinically mild encephalitis/encephalopathy with a reversible splenial lesion (MERS) in corpus callosum. Given the reversibility of the lesions and excellent prognosis, they proposed that this may represent a new clinico-radiological syndrome, in which magnetic resonance imaging (MRI) plays an important role in establishing the diagnosis. From then on, accumulating cases with reversible splenial lesions in the corpus callosum caused by different etiologies have been reported. In 2011, Garcia-Monco et al^[3]^ reviewed the MEDLINE database from 1966 to 2007 and termed the presence of transient lesions involving SCC as reversible splenial lesion syndrome (RESLES). We herein report a case of RESLES associated with lobar pneumonia, and further summarize the up-to date knowledge of RESLES, including the etiology, pathogenesis, clinical manifestations, and radiological features, as well as current treatment and prognosis.

## 2 Case presentation

**Figure 1:**
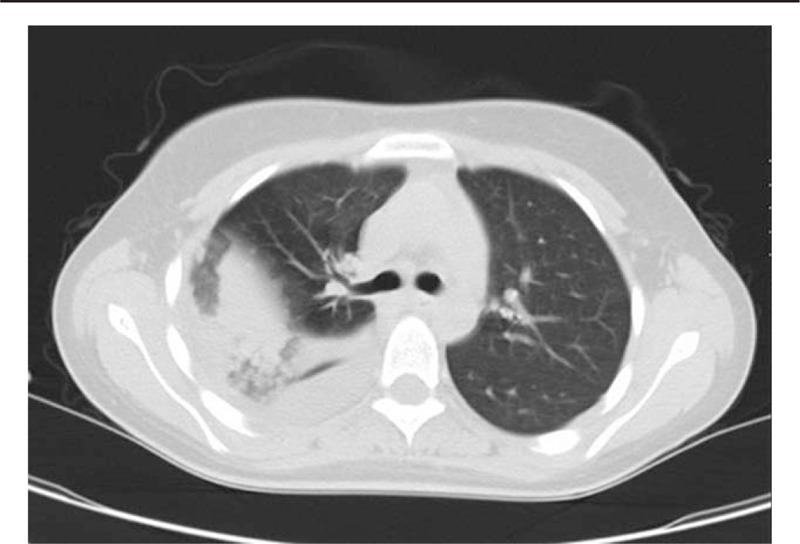
Thoracic computed tomography (CT), thoracic CT of the patient revealed lobar pneumonia.

**Figure 2:**
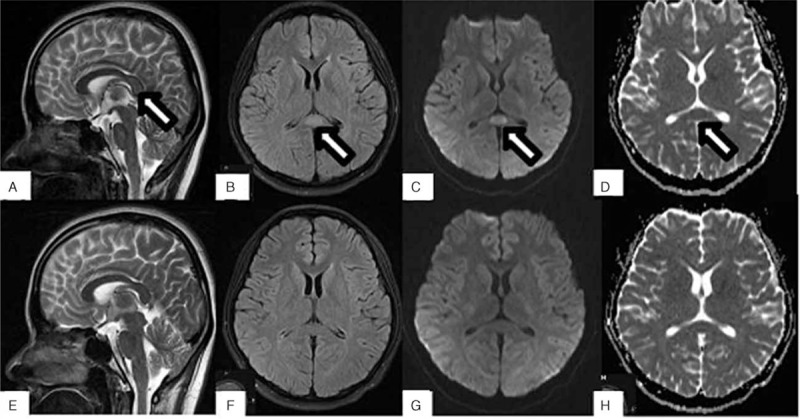
. MRI of RESLES, (A) T2WI, (B) FLAIR, (C) DWI, and (D) ADC showed the lesion in SCC (white arrow) at disease onset. (E–H) Correspond to the follow-up images without any evident lesion. ADC = apparent diffusion coefficient, DWI = diffusion-weighted image, FLAIR = fluid attenuated inversion recovery, MRI = magnetic resonance imaging, RESLES = reversible splenial lesion syndrome, T2WI = T2-weighted image.

An 18-year-old female with a history of long-term dieting was admitted to the Department of Pneumology in the First Hospital of Jilin University with complaints of high fever, cough, rusty colored stuff, and chest pain for 4 days. Inflammation of the pulmonary parenchyma was revealed by a thoracic CT examination (Fig. 1). Antiinfectious treatment with sulbenicillin sodium, moxifloxacin, and vidarabine monophosphate was initiated. *Klebsiella pneumoniae* was found in the sputum 3 days later. Antimycoplasma (MP) pneumoniae IgM antibody in serum was negative. Lobar pneumonia was diagnosed finally combined with the typical clinical manifestations including high fever, cough, rusty colored stuff, and chest pain and the radiological characteristics of inflammation of the pulmonary parenchyma. Five days later, she told her family that some people were on the wall and roof. Meanwhile, she waved her right upper limb without purpose. Then she was transferred to the Department of Neurology. On physical examination, she was fully alert and oriented. Her right upper limb waved irregularly for 15 or 20 times each hour. The tendon reflex of the lower limbs was weakened. The Babinski sign was absent bilaterally. Laboratory investigations including routine blood test, liver, and renal functions revealed slightly increased neutrophilic granulocyte (NE%) (0.77, normal range: 0.4–0.75), hyponatremia (132.8 mmol/L, normal range: 137–147 mmol/L), mildly elevated levels of lactic dehydrogenase (292 U/L, normal range: 135–226 U/L), and α-hydroxybutyrate dehydrogenase (232 U/L, normal range: 78–182 U/L). Reexamined the anti-MP pneumoniae IgM antibody in serum was positive (1:160) after she developed mental abnormalities. The Mantoux test was negative. The results of cerebrospinal fluid (CSF): the level of protein was 0.23 g/L (normal range: 0.15–0.45 g/L), white blood cell was 3×10 ^6/L (0–8 × 10 ^6/L), glucose was 3.76 mmol/L (2.3–4.1 mmol/L), and the culture of CSF was negative. Examination and electroencephalogram were normal. Cranial MRI was performed immediately after she presented with mental abnormalities. A focal splenial lesion in the corpus callosum was identified, which presented as homogeneous hypointensity on T1 weighted imaging (T1WI) and hyperintensity on T2-weighted imaging (T2WI) and fluid-attenuated inversion recovery (FLAIR) (Fig. 2). The lesion appeared hyperintense on diffusion-weighted imaging (DWI) and showed restricted diffusion on apparent diffusion coefficient (ADC) map (Fig. 2). The patient's psychiatric symptoms were significantly improved after 1-week antiinfection and nerve-nurturing treatment with trivitamin B and Xingnaojing for the neurological symptoms. A follow-up examination of cranial MRI (Fig. 2) 15 days later revealed complete resolution of the lesion. Based on the clinical manifestation, laboratory examination, and serial MRI findings, the diagnosis of RESLES was established.

## 3 Discussion

Presence of reversible lesions of the SCC, so alleged RESLES, has been found in patients with a broad spectrum of diseases and clinical conditions. The diagnosis of our case predominantly depends on the reversible clinical symptoms and MRI findings. Although both anti-MP pneumoniae IgM in serum was positive, and *Klebsiella pneumoniae* was found in the sputum. The anti-MP pneumoniae IgM antibody in serum was negative when she was admitted to the hospital. After she developed mental abnormalities, the anti-MP pneumoniae IgM antibody in serum reexamined was positive (1:160). So, we speculated that the pneumonia was due to *Klebsiella*. However, medications that were used in the treatment are not completely exempt from suspicion. Doxycycline was administered immediately after anti-MP pneumoniae IgM was found positive in serum. Vidarabine was discontinued when virus antibody in serum was found negative. One adverse effect of moxifloxacin and doxycycline, in particular, is psychiatric symptom.^[4]^

We searched Pubmed database with the search terms “reversible splenial lesion syndrome” or “RESLES” or “clinically mild encephalitis/encephalopathy with a reversible splenial lesion” or “MERS,” and 502 patients with RESLES comprised in 100 articles were reviewed.

**Figure 3:**
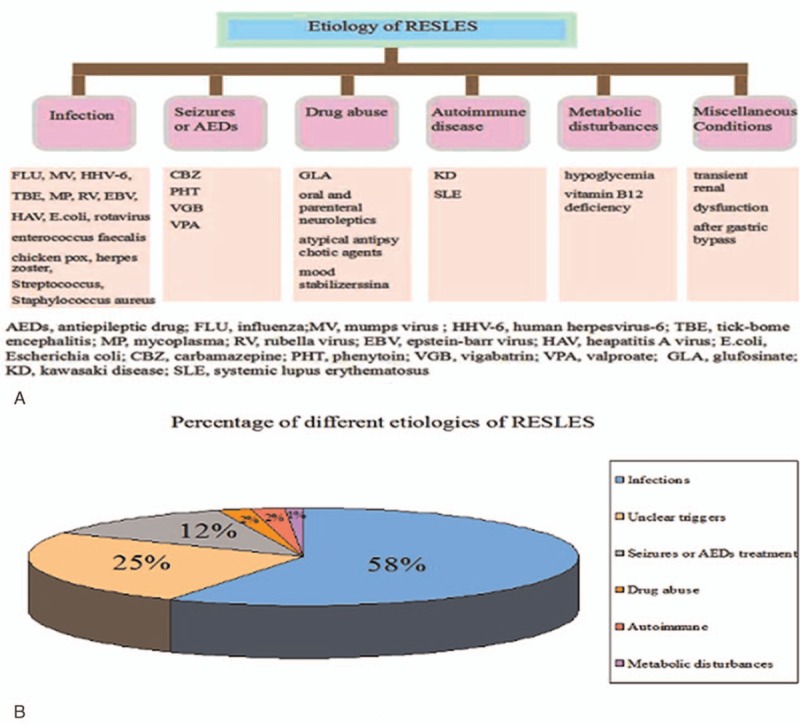
Etiologies and pathogenesis of reversible splenial lesion syndrome (RESLES), possible etiologies and pathogeneses of RESLES are summarized in (A), and the percentages of different etiologies of RESLES are described in (B).

Although the pathogenesis of RESLES remains incompletely clear thus far, increasing cases of RESLES with various etiologies have been reported. Reported etiologies of RESLES are illustrated in Fig. 3. A majority of these cases (292/502, 58%) developed RESLES after infections. Among the remaining patients, 123 (25%) patients developed RESLES with unclear triggers, 62 (12%) patients were triggered by seizures or AEDs treatment, 10 (2%) patients after drug abuse, 9 (2%) patients followed by autoimmune diseases, and 6 (1%) patients due to metabolic disturbances.

Viral infection is the most common reason of RESLES, which includes rotavirus, adenovirus, mumps virus, Epstein–Barr virus, chicken pox, herpes zoster, *Streptococcus*, *Escherichia coli* (*E coli*), *Staphylococcus aureus*, MP, tick borne encephalitis, human herpes virus-6, and hepatitis A virus, etc. (Table 1).^[2–3,12,26,29–34]^ However, the exact pathogenesis of RESLES following infections is still controversial. A large body of evidence suggests that the manifestations of RESLES are related to inflammatory factors, such as interleukin 6, tumor necrosis factor-α, and soluble tumor necrosis factor receptor 1.^[25,26]^ Indeed, a strong correlation between plasma level of interleukin-6 and clinical severity and prognosis of RESLES was reported. However, Miyata R et al found that the levels of inflammatory cytokines like interleukin-6, tumor necrosis factor-α, and soluble tumor necrosis factor receptor 1 during the acute phase of RESLES were not increased.^[99]^ Thus, they speculated that the splenial lesions in the corpus callosum were not induced by neurotoxic cellular inflammatory cytokines. Possible reasons for the transiently reduced diffusion within the lesions have been postulated to include intramylinic edema, interstitial edema in tightly packed fibers, and transient inflammatory infiltration.^[12]^ Miyata et al^[13]^ suggested that oxidative stress may be involved in the pathogenetic process of RESLES. They found that Tau protein and cytokines in CSF of patients with RESLES were increased, and they considered that the change of local osmotic pressure caused oxygen stress response. Brain white matter myelin protein was destructed finally. Patients with infection may be associated with hyponatremia.^[29,31]^ When hyponatremia occurs, the osmotic pressure of the cells in the corpus callosum is decreased and the increased free water entered into the cells leading to cytotoxic edema eventually.^[15]^ Tani et al^[96]^ reviewed clinical date of 24 patients with RESLES and found that all of them had fever, suggesting that fever may be associated with RESLES. The mechanism of fever to result in RESLES may be that fever increased the consumption of brain glucose, thereby reducing local effective osmolarity of brain tissue.^[17]^

**Table 1:**
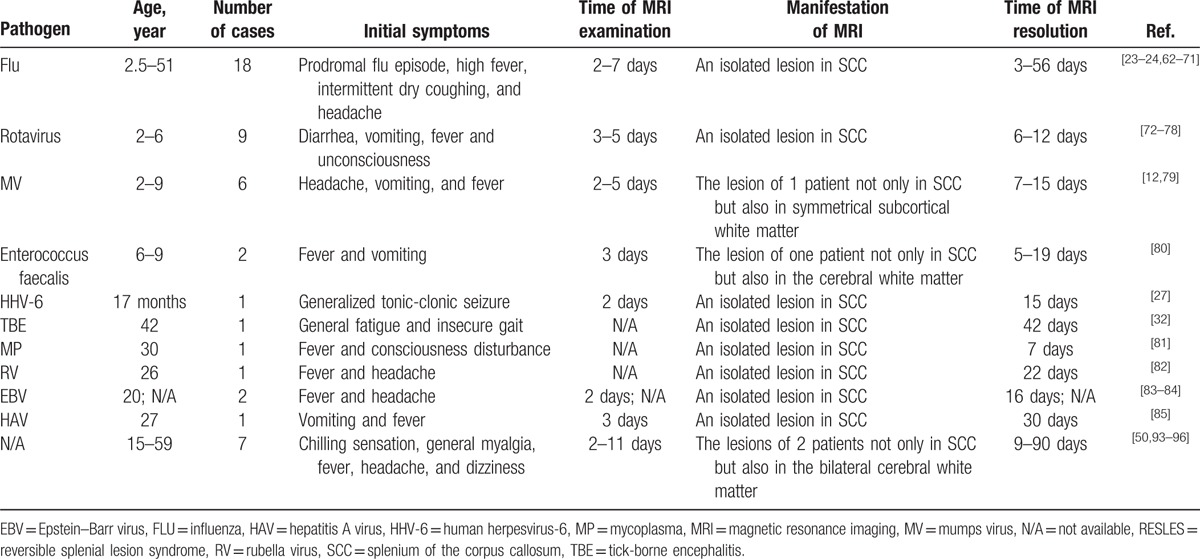
Cases of RESLES following infections.

Kim et al^[1]^ first reported a reversible lesion of the SCC in epileptic patients receiving AEDs. Then growing cases of RESLES has been found in patients with epilepsy or those receiving AEDs (Table 2). Characteristics of these MRI lesions were homogenously reduced diffusion as revealed by DWI and ADC map; however, these lesions were distinct from irreversible cytotoxic edema usually seen in cellular energy failure, such as acute ischemic infarction.^[97]^ Epilepsy is characterized by a group of abnormal discharge of brain neurons in the central nervous system (CNS) function of sudden recurrent and transient disorder clinical syndrome. Öztoprak et al^[5]^ had proposed a possible mechanism of RESLES in which the free water dispersion of the corpus callosum was decreased when the abnormal discharge of corpus callosum is disseminated. Seizures were not considered as an underlying cause of the lesions, although that possibility could not be excluded. Both hyponatremia and seizures might contribute to the evolution of the RESLES. One possible explanation for RESLES associated with AEDs is osmotic demyelination caused by fluid imbalance, including the alternations of sodium and glucose concentration.^[29]^ An osmotic effect might be primarily caused by hyponatremia or other solute imbalances which play an important role in the pathogenesis. However, some authors believe the changes in the concentration of AEDs contributed to the pathogenesis of RESLES.^[2,6–10]^ Polster et al^[9]^ proposed a hypothesis that a transient imbalance in arginine vasopressin (AVP) secretion was associated with rapid concentration changes of AEDs in blood.^[11]^ Water in the brain tissue could be influenced by AVP which regulates regional cerebral blood flow and accounts for brain edema.^[9,14,16,18,19]^ It was also reported that patients receiving carbamazepine for the treatment of trigeminal neuralgia without hyponatremia developed RESLES by resetting osmoreceptors and increasing renal sensitivity to play an antidiuretic effection.^[20,21,22,93]^ Further studies are needed to confirm this hypothesis.^[6]^

**Table 2:**
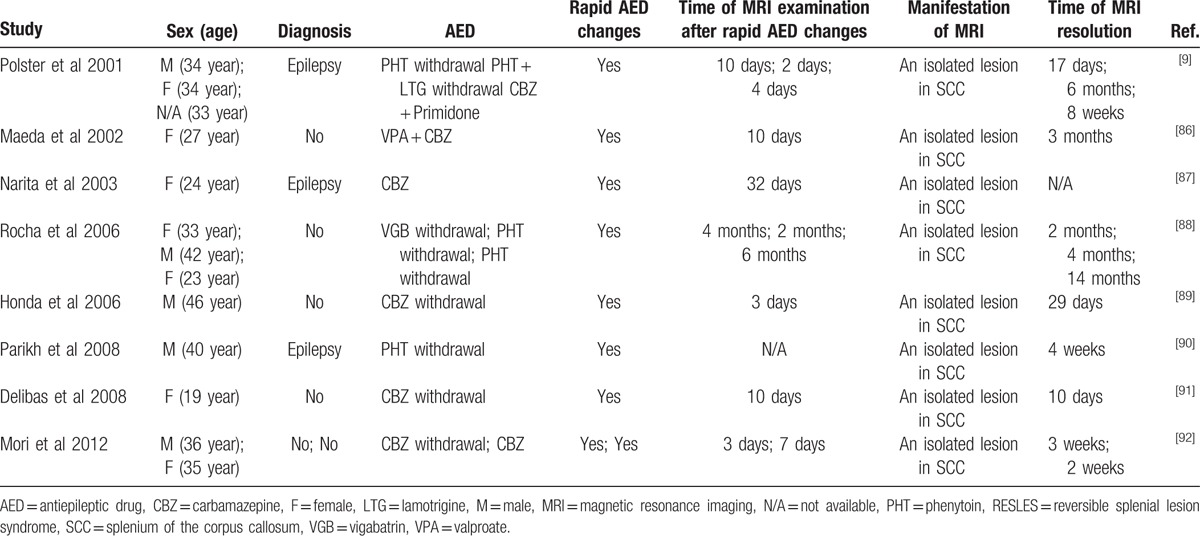
Cases of RESLES following AEDs.

Colleagues from South Korea had reported a 39 year-old woman, who attempted to suicide by taking 200 mL BASTA (18% glufosinate ammonium [GLA]) and then showed reversible splenial lesions in the corpus callosum on MRI.^[33]^ GLA could increase the extracellular glutamate via inhibiting glutamine synthetase in the brain.^[36]^ Additionally, GLA is a structure analog of glutamate which could directly stimulate glutamate receptors. Collectively, the excessive glutamate causes cell death or cytotoxic edema by binding to the *N*-methyl-d-aspartate (NMDA) receptors and non-NMDA receptors.^[35]^ Glutamate also promotes cytotoxic edema by sodium (Na^+^) and calcium (Ca^2+^) entering glial cells and myelin sheath which results in water diffusion eventually. Excitotoxicity and edema of astrocytes and myelin can prevent acute-phase neuronal damage because astrocytes and myelin have glutamate receptors and transporters. This may account for the reversibility of the edema.^[3]^ Achalia and Andrade^[37]^ presented a patient with bipolar disorder who developed an asymptomatic splenial lesion in the text of probable neuroleptic malignant syndrome (NMS). Although RESLES in NMS has been reported previously, there is no sufficient evidence to support that the reversible abnormal signal of the corpus callosum is related to NMS.

Mucocutaneous lymph node syndrome, also called Kawasaki disease (KD), is a kind of systemic vasculitis which usually occurs in pediatric patients.^[39,40]^ At present, KD is considered to be immune-mediated vasculitis. Takanashi et al^[38]^ reported 4 patients developed RESLES with KD. Researchers have found that the mechanism of RESLES in KD patients was incompletely clear.^[38]^ It had been found many proinflammatory cytokines and chemokines were elevated in patients with KD during the acute phase, such as tumor necrosis factor-α, interleukin 1, 6, 8, and vascular endothelial growth factor.^[41–42]^ Proinflammatory cytokines and chemokines might contribute to the inflammatory infiltration which serves as a possible mechanism for transient restricted diffusion in cases of RESLES.^[2]^ Elevated vascular endothelial growth factor could result in hypoalbuminemia, vascular leakage, and noncardiac edema.^[41]^ Electrolyte or water imbalance could lead to cerebral edema and cerebral edema could possibly progress to RESLES. Additionally, systemic lupus erythematosus (SLE) is a kind of autoimmune inflammatory connective tissue disease involving multiple organs in young women. Appenzeller et al^[42]^ reviewed the MRI results of 115 patients with SLE and identified 3 patients with RESLES. Possible pathogenesis is that vasogenic or cytotoxic edema may occur in SLE patients in different disease phases.^[42]^

RESLES has also been reported in patients with hypoglycemia,^[3]^ and those with long-term malnutrition. Long-term malnutrition could cause vitamin B12 deficiency. Vitamin B12 mediates 2 kinds of important enzymatic reactions: methyl malonic coenzyme A converts to succinyl coenzyme A and participates in methionine-homocysteine (Hcy) metabolism.^[43]^ As the cofactor of transmethylase, vitamin B12 participates in the reaction of transferring methyl from methyl tetrahydrofolate to Hcy to synthesize methionine. S-Adenosyl methionine, as a methyl donor, is involved in the methylation process in many important substances, including deoxyribonucleic acid, ribonucleic acid, protein, myelin, and many neurotransmitters. Once vitamin B12 is lacking, formation of S-Adenosyl methionine would be blocked which leads to serious metabolic disorders including nerve myelination disorder and depigmentation.^[44]^ The disorder of myelination leads to demyelination of corpus callosum finally. In addition, accumulating Hcy has cytotoxic effect by stimulating NMDA receptors and activating apoptosis associated protein Bax and P53. Landais^[45]^ has reported 2 patients of RESLES with hypoglycemia. Although the specific mechanism was not clear, several possible mechanisms have been proposed including intramyelinic edema, inflammatory infiltration, and perturbed cellular fluid mechanism.^[46]^ Our patient had a history of dieting which may contribute to her infection and RESLES.

A 6-month-old boy with a transient renal dysfunction was found abnormal in the corpus callosum, and the lesion disappeared after 21 days.^[47]^ The authors speculated that the lesion may be related to the upregulation of cytokines.^[47]^ Elevated cytokines might activate the intracerebral immune response through the immune-neuroendocrine pathway, which played a crucial role in RESLES. Additional studies are required to clarify the mechanism of the upregulation of cytokines with transient renal dysfunction in the development of RESLES. Theeler et al^[48]^ found that bilateral optic neuropathy and RESLES appeared after gastric bypass surgery in another patient. A common pathophysiology may be shared in the patient.^[48]^ RESLES was also found in 2 sisters, and their MRIs revealed reversible lesions in the SCC with lateral extension to the callosal radiations and the frontoparietal white matter. Thus, genetic factors may be involved in the pathogenesis of RESLES,^[49]^ which still need further validation.

RESLES has been associated with various disorders including infection, seizures and/or AEDs, drugs abuse, autoimmune disease, metabolic disturbance, and miscellaneous conditions.^[98]^ Pathogenic mechanism of RESLES is caused by multiple reasons including inflammatory infiltration, fluid imbalance, AVP function disorder and intramylinic edema, and so forth. All of them eventually lead to cytotoxic edema. However, it remains unknown why the splenium is exclusively involved in most circumstances. It may be related to the characteristic of structure and blood supply of SCC. Corpus callosum is one of the largest joint fibers in the brain. Compared with the surrounding tissue, the direction of the fibers is consistent and the fibers are strongly packed together. The myelin sheath of SCC has more water that is uneven than the surrounding tissue.^[97]^ The mechanism of self-protection of the electrolyte imbalance may be insufficient, so the SCC can be more prone to cytotoxic edema than the other parts of the brain.^[28]^ In addition, the corpus callosum department is mainly supplied with vertebral-basilar artery. Although, other parts of the corpus callosum including the rostrum, the knee, and the body are mainly supplied with anterior circulation, including anterior cerebral artery, anterior communicating artery, and pericallosal artery, where the blood supply is more abundant. Thus, the damage of SCC may be related to the structure characteristics of the corpus callosum and the feature of blood supply.

Corpus callosum is estimated to contain 1 million fibers. Callosal commissural fibers could transfer lateralized cortical activities to the contralateral side, and link 2 hemispheres to coordinate bilateral movement, sensation, and visual function. The SCC is located at the back end of the corpus callosum and it plays an important role in the function of transmitting and intergration of writing, objects, face, and visual information. So, when damaged consciousness disturbance, abnormal behavior and so forth may appear. Patients with RESLES will be accompanied with different clinical manifestations under different conditions. We have calculated the frequency of appearance of each of the systemic symptoms and the neurological ones, respectively. RESLES with infection usually following prodromal illness that consisted of fever (38/49, 42.7%), vomiting 15 (16.8%), headache 13 (14.6%), diarrhea and abdominal pain 8 (9.0%), cough 4 (4.5%), arthralgia 3 (3.4%), myalgia 3 (3.4%), rhinorrhea 3 (3.4%), and nausea 2 (2.2%). Consciousness disturbance (223/403, 55.3%) was the most common symptoms of the CNS. Besides, abnormal behavior 40 (10%), seizures 29 (7.2%), irritable and agitation 23 (5.7%), refusal to talk and eat 21 (5.2%), visual hallucinations 20 (5.0%), alert mental status 16 (3.9%), dysarthria 12 (3.0%), bradypsychia 8 (2.0%), disoriented 6 (1.5%), gait ataxia 3 (0.7%), and with incoherent language 2 (0.5%) may also appear.^[50]^ Of note is that the symptoms of the CNS in a majority of patients could resolve completely in 1 month.

The diagnosis of RESLES mainly depends on the MRI findings. The lesion is usually oval with clear boundary whereas without obvious edema and space-occupying effect.^[51]^ On MRI, the lesion is homogeneously hypointense signal on T1WI while hyperintense on T2WI and FLAIR. It appeared hyperintense on DWI and showed restricted diffusion on ADC map. It is noteworthy that the enhanced MRI scan is usually without enhancement effect. In addition, diffusion tensor imaging (DTI) is a promising MRI technique based on fiber tracking during the “lesional” phase, and it shows symmetrical and integral fibers in the posterior aspect of the corpus callosum. DTI including fully automated quantitative fractional anisotropy analysis was used to get insight into the pathophysiology of RESLES. DTI has also shown that a reversible loss of directional fiber organization, indicating a myelin lesion. In combination with the DWI data, it suggests that the loss of directional fiber organization in RESLES might be caused by intramyelinic cytotoxic edema. Significantly loss of directional organization of the central myelin as detected by DTI is reversible.^[52]^

**Figure 4:**
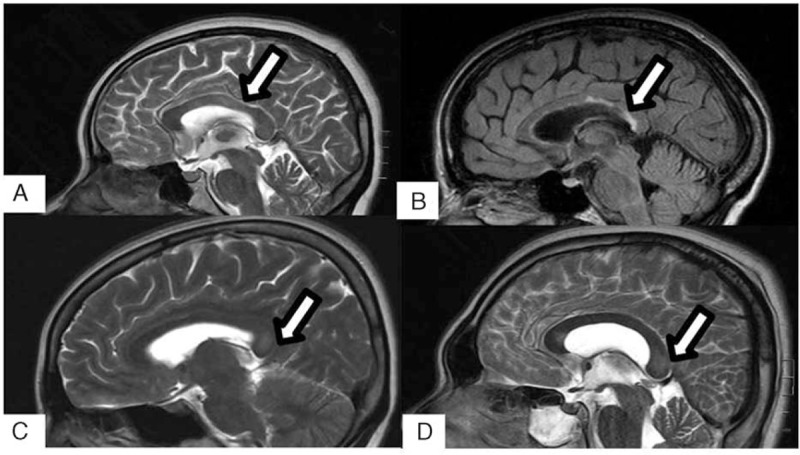
Differential diagnoses of RESLES, (A–D) showed infarction, MS, MBD, and DAI in the splenium of corpus callosum (white arrows). DAI = diffuse axonal injury, MBD = Marchiafava–Bignami disease, MS = multiple sclerosis, RESLES = reversible splenial lesion syndrome.

The neuroimaging changes of the corpus callosum should be differentiated from other pathological lesions, such as posterior reversible encephalopathy syndrome (PRES), infarction of corpus callosum, acute disseminated encephalomyelitis (ADEM), multiple sclerosis (MS), Marchiafava–Bignami disease (MBD), Susac syndrome, corpus callosum injury, tumor, etc. PRES is associated with white matter changes predominantly affecting in posterior parietal and occipital lobes of the brain in a pregnant or puerperal woman. The typical features of PRES on MRI commonly appear as hyperintensity on T2WI and FLAIR images in the parieto-occipital and frontal cortical and subcortical white matter.^[54]^ Infarction of corpus callosum is occasionally seen. Common lesions of callosal infarction are localized on the knee and body of corpus callosum. Moreover, the edge of the lesion in infarction is fuzzy, usually along with other infarction (Fig. 4A).^[53]^ As to ADEM, multiple points and patchy abnormal signals are visible. In addition, lesions usually involve the basal section, thalamus, and corpus callosum body with enhancement of lesions in patients with ADEM.^[55]^ Demyelinating lesions may involve the corpus callosum body and knee with enhancement in the acute phase; while, corpus callosum often undergoes atrophy with focal necrosis in the chronic phase.^[55]^ The typical demyelinating lesions of MS are located in periventricular, juxtacortical, infratentorial, or spinal cord. The plaques lesion of MS in corpus callosal demonstrate contrast enhancement, hyperintensity on T1WI (Fig. 4B), increased DWI and ADC signals in the acute phase and remission relapse of demylination.^[56]^ MBD is a rare disease in patients with chronic alcoholism or nutritional susceptibility and is characterized by symmetric demyelination and necrosis of the corpus callosum which was first described in 1903.^[57]^ The lesions in cranial MRI of MBD are not restricted to the corpus callosum, but may also occur in extracallosal white matter and cortical regions.^[58]^ During the acute phase, the corpus callosum appears hypointense on T1WI, hyperintensity on T2WI (Fig. 4C). Signal intensity alterations became less evident but residual atrophy of the involved structure was seen during the chronic phase.^[59]^ Susac syndrome is a rare immune-mediated endotheliopathy typically resulting in occlusion of the microvasculature in the retina, inner ear, and brain.^[60]^ The typical cranial MRI showed extensive lesions involve corpus callosum, centrum semiovale, internal capsule and thalamus, etc.^[60]^ A 30-year-old man sustained traumatic brain injury (TBI) and subarachnoid hemorrhage secondary to a traffic accident. An examination of cranial MRI 11 days later revealed a lesion in the corpus callosum, which presented as slight hyperintensity on T2WI (Fig. 4D). Corpus callosum is the largest commissural white matter bundle in the brain and is also one of the predilection sites of TBI. Diffuse axonal injury is an important aspect of TBI pathology which is describes a process of widespread axonal damage in the aftermath of acute or repetitive TBI, leading to deficits in cerebral connectivity that may or may not recover over time.^[61]^ We ascribe the lesion to diffuse axonal injury. Unlike RESLES, neoplastic lesions present as hypointense or mixed signal on T1WI, hyperintensity on T2WI, and FLAIR of cranial MRI. The lesions have or not enhancement, and the form of enhancement is variform, including ring form, strip line, schistose, and irregular shape. Neoplastic lesions usually present with mass effects.^[62]^

RESLES is a clinico-radiological syndrome with unclear pathogenesis which could be triggered by different factors. At present the diagnosis and treatment of RESLES, there is no unified standard. The diagnosis of RESLES mainly depends on nonspecificity clinical manifestations and distinctive radiological characteristics. Usually, RESLES does not require special management, the clinical manifestations and lesions on MRI could disappear naturally after 1 month. Patients with RESLES usually have a good prognosis.
